# Novel Micro-Ribonucleic Acid Biomarkers for Early Detection of Type 2 Diabetes Mellitus and Associated Complications—A Literature Review

**DOI:** 10.3390/ijms26020753

**Published:** 2025-01-17

**Authors:** Sara Ahmed, Haroon Adnan, Maryam A. Khawaja, Alexandra E. Butler

**Affiliations:** 1 School of Medicine, Royal College of Surgeons in Ireland-Bahrain, Busaiteen 15503, Bahrain; 20204065@rcsi-mub.com (S.A.); 19200140@rcsi-mub.com (H.A.); 18211593@rcsi-mub.com (M.A.K.); 2 Research Department, Royal College of Surgeons in Ireland-Bahrain, Busaiteen 15503, Bahrain

**Keywords:** type 2 diabetes mellitus (T2DM), prediabetes, biomarkers, microRNA (miRNA)

## Abstract

Type 2 diabetes mellitus (T2DM) is one of the most widespread chronic diseases globally, with its prevalence expected to rise significantly in the years ahead. Previous studies on risk stratification for T2DM identify certain biomarkers, including glycated hemoglobin (HbA1c), oral glucose tolerance testing (OGTT), fructosamine, and glycated albumin, as key indicators for predicting the onset and progression of T2DM. However, these traditional markers have been shown to lack sensitivity and specificity and their results are difficult to analyze due to non-standardized interpretation criteria, posing significant challenges to an accurate and definitive diagnosis. The strict measures of these traditional markers may not catch gradual increases in blood sugar levels during the early stages of diabetes evolution, as these might still fall within acceptable glycemic parameters. Recent advancements in research have suggested novel micro ribonucleic acid (miRNA) as circulatory molecules that can facilitate the early detection of prediabetic conditions in high-risk groups and potentially enable prevention of the progression to T2DM. This capability makes them a very powerful tool for potentially improving population health, enhancing outcomes for many patients, and reducing the overall burden of T2DM. These promising biomarkers are small, noncoding RNA involved in the regulation of many cellular functions that have a hand in the metabolic activities of cells, making them a very useful and relevant biomarker to explore for the diagnosis and risk stratification of T2DM. This review analyzes the current literature, outlining the occurrence of miRNAs in prediabetic and diabetic individuals and their implications in predicting dysglycemic disorders.

## 1. Introduction

Across the spectrum of diabetes, two primary forms are the most prevalent: type 1 diabetes (T1DM) and type 2 diabetes (T2DM). T1DM is an autoimmune disorder characterized by the self-destruction of pancreatic β-cells and is relatively less prevalent than T2DM [1]. Conversely, T2DM is marked by hyperglycemia caused by the loss of β-cells through apoptosis, leaving a residual population of dysfunctional β-cells. Over time, these dysfunctional β-cells cannot counteract the effects of increasing insulin resistance, leading to inadequate insulin secretion and elevated blood glucose levels [2,3,4]. Prolonged hyperglycemia can lead to severe diabetes-related complications, specifically microvascular complications, such as retinopathy, neuropathy, and nephropathy [5,6], or macrovascular complications, such as atherosclerosis, in the vessels supplying the heart, brain, limbs, and other organs.

Before the onset of fully developed T2DM, many individuals pass through an intermediary stage known as prediabetes. Prediabetes is the stage of abnormal glucose homeostasis where blood glucose levels surpass normal glycemic parameters but remain below the diagnostic threshold [7]. Prediabetes is widespread, particularly among older and obese demographics, often persisting for years before the full clinical condition develops [8].

Addressing this early stage is crucial because although pharmacological interventions can slow the progression of T2DM, they do not inherently restore normal metabolic function, leaving patients vulnerable to microvascular complications [9]. Lifestyle modifications represent a non-pharmacological approach well-integrated into clinical medicine for T2DM management [10,11]. Unfortunately, the long-term sustainability of these modifications is compromised by various factors, with patient non-compliance being a prominent issue [12]. Given the growing public health concern of T2DM, with the International Diabetes Federation predicting an increase in prevalence to 400 million cases by 2040, early detection and effective management strategies are essential [13,14].

Diagnosing T2DM heavily relies on biomarkers, which are objective and quantifiable measures showing the biological and pathological processes occurring within organisms [15]. Conventional biomarkers have undergone extensive research and have become established in clinical medicine as standard diagnostic tools [16,17]. However, emerging biomarkers, including proteomic and micro ribonucleic acid (miRNA) markers, have yet to be validated in clinical settings.

Epigenetic mechanisms, including DNA methylation, histone modification, and ncRNAs, regulate gene expression without altering the underlying DNA sequence. Epigenetics represents a rapidly emerging field of research with significant implications for understanding the heritability and progression of various diseases, including T2DM [18]. Among the various aspects of epigenetics, noncoding RNAs (ncRNA) are increasingly recognized for their pivotal role in orchestrating cellular function. MiRNAs, a subset of ncRNAs, are particularly significant due to their capacity to regulate gene expression post-transcriptionally [19,20]. MiRNAs’ ability to regulate genes allows them to control several biological and pathophysiological processes, including cell death and proliferation [21]. The dysregulation of miRNA can disrupt signaling pathways, as is observed in T2DM, resulting in disease development and progression through interference with the critical insulin signaling pathways [22].

This literary analysis first examines well-established traditional biomarkers and then considers more recently identified novel miRNA biomarkers closely associated with T2DM and their potential application in clinical medicine to facilitate the early detection of prediabetic and T2DM patients. Various microRNAs have been reported to be associated with diabetes. The miRNAs selected for this paper were reported in greater frequency in diabetic patients (miR-375 and -126, 7a, and Let-7) and diabetic complications (miR-373, -181B, -21, -192, 133b, 342-3p, and -30), highlighting their potential diagnostic value in diabetes.

## 2. Traditional Biomarkers

### 2.1. HbA1c

Hemoglobin A1c (HbA1c), an essential component in clinical diagnostics, is formed by gradually incorporating glucose molecules into the amino-terminal group of the hemoglobin beta subunit within the circulatory system [23]. The HbA1c level indicates an individual’s glycemic control over the preceding three months [24]. The criteria outlined by the American Diabetes Association (ADA) and the National Institute for Health and Care Excellence (NICE) in the United Kingdom report HbA1c values of ≥6.5% (48 mmol/L) for diabetes and ≥5.7% to 6.4% (39 to 46 mmol/L) for prediabetes [1,25]. HbA1c eliminates the need for fasting, offering a more patient-friendly assessment process [26]. It also has a lower biological variability and is unaffected by short-term disturbances, thus ensuring a stable pre-analytical phase [26,27]. On the other hand, HbA1c may not be optimal for early diabetes detection [28]. Particularly during the prediabetic phase, during which blood glucose levels experience minor elevations, HbA1c may remain <5.5% (37 mmol/L), potentially failing to detect subtle changes in glucose levels accurately based on ADA guidelines [29,30]. Additionally, clinical conditions that reduce (iron deficiency anemia) or promote red blood cell turnover (splenomegaly) can introduce inaccuracies in HbA1c measurements [31,32,33]. Other states, including severe hyperbilirubinemia (>20 mg/dL) and severe hypertriglyceridemia (>1750 mg/dL), can also contribute to inaccurate elevations in HbA1c levels [34].

### 2.2. Fructosamine

Fructosamine is an alternative method for monitoring glycemic control in T2DM and holds potential for the diagnosis of prediabetes [35]. This biomarker results from the glycation of circulatory proteins, including albumin, globulins, and lipoproteins. Increased glucose concentration in the blood causes increased binding to serum proteins, with a greater affinity for albumin [36]. Fructosamine can be used alongside HbA1c in T2DM because it reflects the glucose profile for 2 to 3 weeks before the test [36,37]. Fructosamine, however, has limitations. Conditions affecting albumin levels, such as liver or kidney disease, can alter fructosamine results [35]. These conditions lead to a rapid protein turnover, resulting in falsely low fructosamine levels [38]. Furthermore, fructosamine does not account for individual variations in albumin metabolism, a process influenced by age, sex, and environmental variables [39,40].

### 2.3. Glycated Albumin

Glycated albumin (GA) is a product of the non-enzymatic glycation of albumin that occurs in the bloodstream. This process results from the reaction between glucose and albumin, in which glucose binds to the amino groups of albumin, forming a ketoamine [41]. The rate of albumin glycation depends on blood glucose levels and the duration of albumin’s presence in the bloodstream [42]. The reference ranges for normal albumin levels in an adult’s blood range from 3.5 to 5.5 g per deciliter (g/dL) [43]. Ongoing studies have suggested that GA levels above 16–17% may indicate poor glycemic control, but these values can vary depending on the individual’s health status [44,45,46]. Despite its clinical utility, glycated albumin has limitations that warrant consideration. Factors such as hypoalbuminemia, liver disease, and inflammatory conditions can influence glycated albumin measurements [47,48,49]. Additionally, the need for standardized reference ranges for glycated albumin poses challenges in interpreting results consistently across different laboratories and patient populations.

### 2.4. Oral Glucose Tolerance Test

The Oral Glucose Tolerance Test (OGTT) ascertains whether a patient’s body can effectively use and store glucose [50]. It is often utilized to test for conditions such as diabetes mellitus or insulin resistance and may assess pancreatic β-cell function impairment [51,52]. An OGTT begins with an initial measurement of the patient’s fasting plasma glucose (FPG) level. The reference ranges for FPG levels are as follows: non-diabetic < 100mg/dL (<5.6 mmol/L), prediabetic 100–125 mg/dL (5.6–6.9 mmol/L), and diabetic ≥ 126 mg/dL (≥7 mmol/L). Ideally, the test is performed when the patient’s pretest FPG levels are between 100 and 125 mg/dL (5.6–6.9 mmol/L) [53,54]. Blood glucose ranges and interpretations following the OGTT are outlined in Table 1. Impaired glucose tolerance is considered when the 2 h 75 g OGTT yields range between 140 and 199 mg/dL [53,55].

While the OGTT is a valuable diagnostic tool, it has limitations. OGTT procedures are expensive and often time-consuming. Additionally, the preparations expected from the patient, such as diet, activity, and stress level, can affect the results [55]. Furthermore, OGTT results may be difficult to interpret in cases where the readings are borderline [52]. In 20% of cases, OGTT results may be categorized as non-diagnostic due to significant differences in glucose concentrations between the samples [56].

**Table 1:** miRNA expression and targets in type 2 diabetes mellitus.

Biomarker	Expression	Target	Clinical Value	Reference
miRNA-375	Up-/Downregulated	MTPN, PDK1	Dysregulated levels can be detected early compared to traditional biomarkers in serum of high-risk groups. Can be utilized for investigative procedures for early detection of diabetes and prediabetes in high-risk groups.	[57,58,59]
miRNA-126	Downregulated	IRS-1	Investigative utility—may be used as part of screening tool for early detection of diabetes, as well as microvascular and retinal complications. Can further monitor levels to observe response to treatment.	[22]
miRNA-7a	Upregulated	IRS-2	Increased expression observed in the serum aids in identifying dysregulated insulin signaling and may be used as an investigative tool to detect and monitor response to treatment.	[60,61]
Let-7	Upregulated	IGF1R, INSR, IRS-2	Increased levels in the plasma suggest impaired/decreased insulin secretion, a key factor in the pathogenesis of T2DM. Potential to aid in early detection of diabetes in clinic.	[62]

## 3. MicroRNA

MicroRNAs are small noncoding RNA about 22 nucleotides long and endogenously expressed [63]. MicroRNAs can regulate cellular function by suppressing gene expression and have been associated with metabolic activities, cellular development, proliferation, and apoptosis [64].

These molecules are part of an epigenetic regulation network, in which several miRNAs, including low-expressed miRNAs, will interact with the same UTR (3′UTR) and regulate gene expression [65,66]. The interaction of the miRNAs with the 3′UTR of the mRNA target can lead to degradation and blocked translation of the target mRNA [64,67].

MiRNA biogenesis can occur through canonical and non-canonical pathways and primarily involves the co-transcription of RNA polymerase II/III proteins [68]. Sequences from the DNA are transcribed into primary miRNAs, with further transcription into precursor miRNAs, and eventually mature into the miRNA form [68]. Many identified miRNAs are primarily transcribed from intragenic introns but may also be intergenic and processed independently [69,70].

Moreover, the pathological expression of miRNAs can play a role in various disease development and progression, including autoimmune conditions such as T1DM and T2DM [71,72]. MiRNAs often occur in high levels in body fluids such as saliva, urine, and plasma and offer biological and chemical stability, thus allowing for miRNA detection in the clinical setting as biomarkers of various diseases [73].

## 4. MicroRNAs Role in T2DM

Specific miRNAs are suggested to regulate protein-coding genes in the insulin signaling pathway and further mediate insulin-regulated glucose homeostasis [74]. Alteration of miRNA expression can impact glucose metabolism and insulin secretion, further changing the target tissue’s response to insulin and leading to insulin resistance [75]. Insulin resistance is characteristic of T2DM and is seen in response to abnormalities in the signaling pathway due to the dysregulation of key proteins [76,77].

Various miRNAs implicated in glucose homeostasis were shown to be expressed in pancreatic β-cells, skeletal muscles, and serum [78,79,80]. MiRNAs expressed in pancreatic β-cells are suggested to be critical in the regulation and proliferation of β-cell mass [81]. Additionally, miRNAs regulate genes, and the pathological expression of miRNAs will target specific protein-coding genes in the insulin pathway, as seen in Table 2, impacting β-cell survival and insulin secretion [73,82].

### 4.1. miRNA-375

MicroRNA-375 is a circulatory noncoding RNA expressed in glucagon-secreting alpha cells and insulin-secreting β-cells of pancreatic islets [90,91]. MiRNA-375 has a significant role in insulin homeostasis; recent studies have shown that miRNA-375 can repress glucose-induced insulin secretion by targeting the *myotrophin gene* (*Mtpn*), which aids in insulin secretion through exocytosis, and the *3′-phosphoinositide-dependent kinase-1* gene (*PDK1*), which activates the *phosphatidylinositol 3-kinase pathway* (*PIK3*), resulting in β-cell dysfunction and decreased insulin secretion [57,58,92]. The dysregulation of miRNA-375 can affect these target genes and cause the inhibition or overexpression of their pathways [93,94].

Profile studies of miRNA-375 in type 2 diabetic, prediabetic, and non-diabetic patient samples were conducted [59]. Due to miRNA being a circulatory biomarker, blood samples are a sufficient and noninvasive method to collect, isolate, and determine miRNA-375 levels [59].

In T2DM, progressive β-cell dysfunction leading to β-cell destruction is an essential pathophysiological characteristic of disease progression [95,96]. The upregulation of miRNA-375 in T2DM corresponds with decreased β-cell levels [97].

Recent experimental research has observed an increased prevalence of miRNA-375 expression in high-risk patient groups for T2DM onset and those with first-degree relatives with T2DM [59,98]. Wu and colleagues similarly underline the overexpression of miRNA-375 in at-risk patients and their relatives with diabetes [59]. The results from the studies suggest that miRNA-375 is a reliable biomarker for predicting and detecting prediabetes and T2DM [59]. 

Moreover, the upregulation of miRNA-375 has shown a positive association with glycemic control values, including OGTT, FPG, and HbA1c, offering substantial diagnostic ability for prediabetes and T2DM [59].

However, limitations have stalled the incorporation of miRNA-375 into clinical medicine. Supplemental research on larger sample groups considering different demographics is required to validate miRNA-375 as an appropriate biomarker in high-risk groups [59,99]. In addition, further studies of human sample groups are necessary to evaluate whether the dedifferentiation of β-cells is induced by pathologic miRNA-375 upregulation during the development of T2DM [81,100].

### 4.2. miRNA-126

MicroRNA-126 is a small noncoding molecule that is found abundantly in endothelial cells [101]. The function of miRNA-126 includes binding to the vascular endothelial growth factor A (VEGF-A) messenger molecule to regulate and inhibit the VEGF molecule [87,102]. Furthermore, miRNA-126 is critical in angiogenic cell maintenance, regulation, and apoptosis [103]. Pathological downregulation can lead to uncontrolled angiogenesis, which is implicated in T2DM microvascular complications [104]. MicroRNA-126 is a circulatory marker that can be an effective noninvasive tool in detecting conditions of diabetic vascular injury and hyperglycemia [105,106].

In a recent study published by Liu et al., researchers assessed the level of circulating miRNA-126 in four patient groups: newly diagnosed T2DM patients, patients with impaired glucose tolerance (IGT), patients with impaired fasting glucose (IFG), and a control group of healthy individuals. The results indicate a significant decrease in miRNA-126 levels for the T2DM, IGT, and FGT groups compared to their healthy counterparts [107]. The study concluded that a reduction in the expression of miRNA-126 is associated with increased glucose and suppressed insulin secretion [107]. Zampetaki and colleagues similarly noted a significant decrease in miRNA-126 in T2DM patients [72]. Additionally, they detected a gradual decline in miRNA-126 within a two-year follow-up period in patients who presented with normal glucose tolerance and progressed to T2DM [72]. The data collected from these studies underline the viability of miRNA-126 as a predictive tool in the early diagnosis of prediabetes and T2DM.

Additionally, miRNA-126 can be further utilized as a biomarker for diabetic retinopathy [108]. The downregulation of miRNA-126 in endothelial cells reduced the inhibition of VEGF. The increased VEGF molecules are an important factor in the development of retinopathy, contributing to retinal neovascularization [108].

While miRNA-126 has significant prognostic and diagnostic value in prediabetes, T2DM, and microvascular complications, more studies in larger cohorts are still required to clearly define its clinical utility.

### 4.3. miRNA-7a

MicroRNA-7a is highly expressed in the pancreatic islet cells and has been suggested to play an essential role in maintaining β-cell mass and the process of insulin secretion [109]. Previous studies have shown miRNA-7a to be co-expressed with transcriptional factors, including RNA binding protein, and neuronal cells are involved in regulating its expression [110,111].

Prior research on the presence of miRNA-7 in diabetic patients and those with microvascular complications compared to healthy controls has suggested an overexpression of serum miRNA-7 [60,112]. One study showed an increase in serum miRNA-7 of 401 ± 34 fmol/L and 501 ± 82 fmol/L (*p* < 0.001) in patients with T2DM and T2DM with complications, respectively, with the level in the control subjects being much lower at 176 ± 17 fmol/L [60]. 

The upregulation of miRNA-7 dysregulates the insulin signaling pathway by repressing the expression of specific genes, such as the *insulin receptor gene* (*INSR*) and *insulin receptor substrate 2* (*IRS-2*) [109]. However, additional in vivo work found miRNA-7 to be decreased in diabetic mice. Ji et al. investigated the role of miRNA-7 in diabetic retinopathy, with the results showing lower levels of miRNA-7 in the endothelial cells (EC) and retinal pericytes (RP) of diabetic mice and increased levels of *IRS-2* [113]. Cao and colleagues considered the role of miRNA in the expression of *PI3K*, the protein kinase B (AKT) pathway, and the VEGF protein through miRNA-7 mimics and found that the overexpression of miRNA-7 downregulates the expression of *PI3K*, AKT, and the VEGF protein, thereby downregulating retinal cell proliferation [114].

Currently, there are conflicting studies on miRNA-7 expression and its associated effect on diabetes and its complications. While an association with the pathway of T2DM diabetes exists because miRNA-7 plays a regulatory role in insulin secretion, further studies are required to validate the implications of miRNA-7 in the development of T2DM before it can be usefully integrated into clinical medicine

### 4.4. Let-7

Let-7 was one of the first miRNAs to be discovered; it has 12 conserved isoforms and is found in a wide array of organisms [115]. Current research has observed the dysregulation of Let-7 in cancer; Let-7 serves as a tumor suppressor by inhibiting the expression of oncogenes [61,116].

Recent studies have investigated the function of Let-7 miRNA in the pancreatic β cells of mice [117]. Let-7 was found to be a crucial miRNA in regulating insulin secretion and β cell proliferation. Furthermore, the overexpression of Let-7 is suggested to be associated with decreased insulin secretion because of the inhibition of β cell proliferation and decreased cyclin D1 and D2 [117,118]. Additional studies conducted on patients with T2DM and control subjects have reported an increased level of serum Let-7b in the T2DM patient group (*p* < 0.05) [118].

Let-7 is further suggested to be associated with retinal complications. The relevant literature has shown Let-7 to be expressed in endothelial and retinal cells. Studies conducted in diabetic mice showed an upregulation of Let-7 to cause non-proliferative diabetic retinopathy [119,120]. Current studies suggest that Let-7 is a robust biomarker that has the potential to aid in the early detection of T2DM and associated complications such as diabetic retinopathy.

## 5. miRNAs in T2DM-Associated Complications

**Figure 1:**
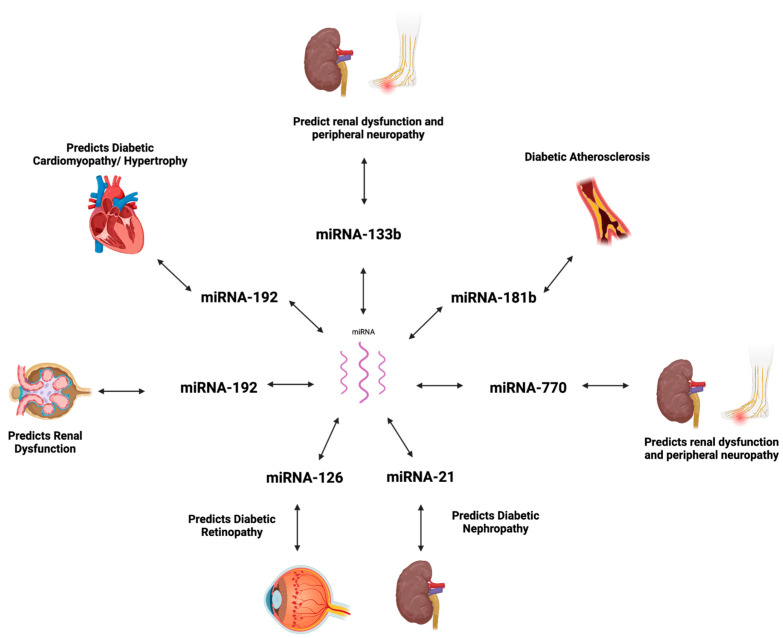
MicroRNA expression in diabetic complications. Created using BioRender.com [122].

Characterized by β cell dysfunction/destruction and insulin resistance, the progression of T2DM can result in various microvascular and macrovascular complications. The pathological expression of miRNAs, which is highlighted in Table 2, is suggested to be implicated in the pathophysiology of vascular complications, such as diabetic retinopathy (DR) and diabetic nephropathy (DN) (Figure 1) [121].

### 5.1. Diabetic Cardiomyopathy

Diabetic cardiomyopathy is a leading complication in diabetic patients. The disease process leading to DC is the result of persistent hyperglycemia in the setting of insulin resistance in cardiac cells, which pushes the myocardial cells to utilize fatty acids, leading to the accumulation of lipids [62]. Eventually, the deposition of lipid intermediates causes myocardial fibrosis and dysfunction [62]. Hypertrophy of the cardiomyocyte in the setting of high glucose has been shown to activate the mitogen-activated protein kinase (MAPK), a signaling pathway that is implicated in inflammation, fibrosis, and the dysfunction of the myocardium [83,123]. MiRNA-373 expression is suggested to be regulated by p38 MAPK, where the inhibition of the signaling pathway results in decreased expression of miRNA-373 [124].

Evidence shows miRNA-373 to have protective effects against hyperglycemia-induced cardiac downregulating of the *myocyte enhancer factor 2C* (*MEF2C*) gene, which codes for the hypertrophic protein [124]. Experiments with diabetic mice revealed downregulated levels of miRNA-373 in the cardiac tissue, which is suggested to be a result of oxidative stress on cardiomyocytes induced by persistent hyperglycemia. Thus, investigating the expression of miR-373 in cardiomyocytes can help in the early detection of cardiac dysfunction and further facilitate early treatment in high-risk groups [124].

### 5.2. Atherosclerosis

Cardiovascular disease and atherosclerosis are significant complications of DM. The progression to atherosclerosis is suggested to be mediated by mechanisms including dyslipidemia, leading to lipid accumulation and the production and accumulation of advanced glycation end products in the setting of hyperglycemia [125,126]. Atherosclerotic plaques consist of macrophages, which induce inflammation and plaque formation. Diabetes-associated atherosclerosis can further be studied using miR-181b, which is suggested to regulate macrophage accumulation that leads to plaque formation [84]. Sun et al. suggested increased miR-181b to have protective effects against atherosclerosis by targeting PHLPP2—a phosphatase that inhibits proliferation cell proliferation [127,128]. An experiment using the endothelial cells from the epididymal white adipose tissue of insulin-resistant mice showed downregulated mIR-181b, allowing for the accumulation of macrophages [128].

### 5.3. Diabetic Nephropathy

DN is a common complication of DM and a leading cause of renal failure [129]. The disease progression of DN is a result of oxidative stress and the release of proinflammatory and fibrotic mediators because of hyperglycemia. Eventually, the release of these compounds causes functional and structural changes to the renal system, leading to interstitial fibrosis and glomerulosclerosis [130]. Significant tissue damage is often present by the time microalbuminuria is detected [129]. Current diagnostic methods, including urine microalbuminuria, may not be adequate in providing an accurate progression of the disease. MiRNA-21 regulates transforming growth factor-β (TGF-β) signaling and is a potential biomarker for DN progression. The overexpression of miRNA-21 may target and suppress *mothers against decapentaplegic homolog family member 7* (*SMAD7*), an inhibitor of TGF-β, ultimately producing fibrotic and inflammatory markers. Identifying increased levels of miR-21 from renal tubular epithelial cell samples in high-risk groups can aid in the easy detection of the potential onset of diabetic nephropathy and guide treatment [131,132]. Moreover, prior research highlights that the upregulation of miRNA-192 targets the repressors *ZEB1* and *ZEB2* and promotes the expression of *Col1a2* and *Col4a1* fibrotic genes [133]. A recent publication identified dysregulated urinary exosomal miRNA-133b, -342-3p, and -30a-5p as viable biomarkers in the pathogenesis of DN, which can also be used as early diagnostic markers [134]. A meta-analysis on the biomarkers of DN reports downregulated urinary values of miRNA-126 and miRNA-770 in diabetic patients, suggesting their involvement in renal disease [135]. 

### 5.4. Diabetic Retinopathy

DR represents a severe consequence of T2DM and a significant cause of blindness in adults between the ages of 20 and 75 years [136]. DR is a progressive disease, with the early stages characterized as non-proliferative (NPDR) and preventable [137]. The late stage is proliferative (PDR) and may subsequently lead to blindness [138]. MiRNA-126 downregulation and reduced VEGF inhibition contribute to the development of retinal damage and DR [106,108]. Additional research outlines the upregulation of miRNA-21 to promote tumor angiogenesis and downstream production of VEGF molecules, contributing to retinal neovascularization and DR [85,139].

## 6. Conclusions

This review of the existing literature underlines the pragmatic benefits of using specific and sensitive microRNA biomarkers in predicting the onset of prediabetes and T2DM during their early stages when β cell function can still be preserved with lifestyle interventions.

While traditional tools for screening and diagnosing dysglycemia, such as HbA1c, OGTT, and GA, are proven to be beneficial, they have limitations and may underestimate the risk of disease progression or even fail to result in a diagnosis. This review highlights the associations of miRNA biomarkers in predicting and diagnosing prediabetic patients and the progression of T2DM. 

The dysregulation of specific miRNAs is often seen in prediabetic and T2DM patients and is consistent with diabetic risk factors and glycemic control values; furthermore, miRNAs can be used to detect associated vascular complications, such as diabetic retinopathy or nephropathy, at an early stage. Therefore, while the dysregulation of these miRNA biomarkers has shown potential in early detection of dysglycemic status, future studies on larger cohorts should focus on comparative analysis and assess their utility in identifying dysglycemic conditions and improving clinical outcomes.

Moving forward, technological advancements have paved the way for newer methods to predict T2DM and its complications. Novel techniques, such as bioinformatics, are currently being explored to identify specific genes and pathways associated with T2DM, as well as proteins that may be expressed or play a role in the pathogenesis of the condition [89].

Researchers have used advanced software and computerized analyses to process and identify microarray data from control and affected patient samples from tissues containing beta cells to isolate and pinpoint specific factors, such as genes, microRNA, and transcription factors, directly linked to the development and progression of T2DM [89]. Another emerging field of research related to microRNA is that of precision medicine. The identification of specific microRNA in individuals will help tailor treatment specific to each case and create a broader spectrum of treatment options and algorithms [120]. Network medicine is another domain that directly relates to identifying disease genes and their interactions to help identify disease pathways and predict other disease genes [140]. Its main aim is to create a diseasome, which is a map of a disease [140]. The map displays multiple diseases as a “node”, and the connections between them display how different pathologies may be related through shared molecular or cellular mechanisms [140]. This methodology is becoming highly relevant for drug design and disease classification, which can further help to create better therapeutics for the complexities and comorbidities associated with T2DM [140].

Finally, another fascinating developing realm is artificial intelligence or machine learning models. Researchers have been using these technologies to enhance the miRNA detection techniques and help to identify comorbidities that may present along with T2DM [141]. With T2DM being such a complex and multifactorial disease, primitive methods have proven to be inaccurate and lack the sensitivity and specificity required for the adequate diagnosis and therapeutics of the disease. In this current age of new technologies, microRNAs have streamlined the process of detection and made it more efficient to predict the development of the disease and its complications. The newer technologies being explored only widen the horizons to create more precise and effective diagnostics and treatments for this highly prevalent disease.

## Figures and Tables

**Table 2:** MicroRNAs and their expressions in complications of type 2 diabetes mellitus.

miRNA	Expression	Clinical Value	Reference
miRNA-373	Decreased expression is linked to hypertrophy mediated by MEF2C.	Diagnostic biomarker of diabetic cardiomyopathy, with dysfunction leading to hypertrophy of cardiomyocyte; early detection of decreased levels in myocardial cells can aid in timely diagnosis in high-risk groups.	[83]
miRNA-181b	Decreased expression is linked with increased macrophage accumulation and pathogenesis of diabetic atherosclerosis.	Decreased expression can be identified in white adipose tissue, allowing early detection to guide diagnosis and treatment.	[84]
miRNA-21	Increased expression is linked to fibrosis and inflammation mediated by TGF-β and suppression of SMAD7.	Detecting upregulated levels in kidney/renal tubular epithelial cells can indicate early stages of diabetic nephropathy, aiding diagnosis, and can further be targeted for therapeutic purposes to decrease fibrosis and inflammation.	[85]
miRNA-192	Decreased expression is linked to decreased urine albumin ratio. It is mediated by TGF-β and is associated with fibrosis.	Levels are decreased in early states of renal injury and can be identified in peripheral blood, allowing viability as an early diagnostic biomarker for diabetic nephropathy.	[86]
miRNA-133b	Dysregulated expression is linked with renal dysfunction and mediates fibrosis in renal tissue.	Dysregulated expression can be detected in the urine in micro or normal albuminuria states, with the potential for use as an early detection tool for diabetic nephropathy and further guiding treatment.	[87]
miRNA-342-3p	Upregulated/downregulated expression is linked with renal dysfunction and associated with injury to the renal tissue.	Altered levels can be identified in urine during early states of albuminuria to aid in the early detection of diabetic renal injury with potential progression to diabetic nephropathy.	[87]
miRNA-30b-5p	Dysregulated expression indicates renal dysfunction and may be used as a biomarker of DN.	Diagnostic urinary biomarker for diabetic nephropathy with potential for early detection of renal injury that can aid in monitoring response to treatment.	[88]
miRNA-126	Decreased expression can serve as a biomarker for DR.	Decreased expression can be identified in serum of prediabetic and diabetic patients for early detection of potential progression to diabetic retinopathy and aid in monitoring response to treatment.	[89]

## Data Availability

No new data were generated in the writing of this review article.
